# Editorial: Heparan Sulfate Proteoglycans and Their Endogenous Modifying Enzymes: Cancer Players, Biomarkers and Therapeutic Targets

**DOI:** 10.3389/fonc.2020.00195

**Published:** 2020-02-21

**Authors:** Cinzia Lanzi, Edwin Alexander Yates, Giuliana Cassinelli

**Affiliations:** ^1^Molecular Pharmacology Unit, Department of Applied Research and Technological Development, Fondazione IRCCS Istituto Nazionale dei Tumori, Milan, Italy; ^2^Department of Biochemistry, Institute of Integrative Biology, University of Liverpool, Liverpool, United Kingdom

**Keywords:** heparan sulfate proteoglycan, heparanase, sulfotransferase, glypican, cancer therapy

Heparan sulfate proteoglycans (HSPGs) are glycoproteins ubiquitously expressed at the cell surface and in the extracellular matrix (ECM). The coordinated action of several heparan sulfate (HS) biosynthetic (e.g., sulfo-transferases) and modifying enzymes (e.g., heparanase, sulfatases) provides these molecules with a marked structural diversity and a peculiar ability to interact with a plethora of biomolecules through HS chains. Dysregulation of HSPGs has been associated with tumor pathogenesis and progression. Moreover, altered expression or deregulated function of HSPG biosynthetic/modifying enzymes has been implicated in key processes including proliferation, angiogenesis, metastasis, and drug resistance. Exploitation of the broad potential of HSPGs and related enzymes as biomarkers and therapeutic targets requires in-depth understanding of the context-dependent and, in some cases, contradictory roles of these molecules in tumors and their microenvironment. This aspect is highlighted by authors in the present Research Topic where mechanistic insights into the multifunctional roles of HSPGs and related enzymes in cancer and immune regulation are provided with a focus on cell signaling, structural issues, and therapeutic implications.

An overview of alterations that characterize the progressive destruction of the normal ECM leading to establishment of a cancer-permissive microenvironment, is provided by Elgundi et al.. Underlying the role of HSPGs in the various stages of the metastatic process, they focus on perlecan, a basal membrane HSPG that may exhibit opposing functions related to either the cellular context or its modular structure (Elgundi et al.).

Special attention has been dedicated to glypicans (GPCs), a subgroup of cell membrane HSPGs predominantly expressed during embryonic development in a strictly regulated way, while being undetectable in most adult tissues. GPCs regulate relevant morphogenic signaling pathways, including those involving Wnts, Hhs, BMPs, and FGFs. Seminal studies in hepatocellular carcinoma (HCC) provided the rationale endorsing the development of GPC3-targeted immunotherapy. GPC3, expressed in over 80% of HCCs, emerged as an actionable therapeutic target as well as useful prognostic biomarker. The review of Kolluri and Ho addresses the role of glypican in regulating HCC cell signaling. GPC3 forms a complex with both Wnt and the Frizzled receptor, activating canonical Wnt**/**β-catenin signaling. Moreover, GPC3 rescues circulating Wnt providing ligand storage at the cell surface. The activity of the 6-O-sulfatase Sulf-2, overexpressed in 60% of HCC, contributes to the release of HS-stored Wnts further promoting Wnt signaling activation. Furthermore, in cooperation with the transcription co-activator Yap, also overactivated in HCC, GPC3 may contribute to the development of liver malignancy by modulating the Hippo pathway (Kolluri and Ho).

Immunotherapeutic strategies developed to target GPC3 include peptide vaccines, immunotoxins, monoclonal and bispecific antibodies, cytotoxic T lymphocytes (CTLs), and engineered T cell therapies. Codrituzumab (GC33), an anti-GPC3 recombinant humanized mAb and ERY974, an anti-GPC3/CD3 bi-specific T cell-redirecting antibody, are currently under clinical evaluation in HCC patients. Shimizu et al., report results from early clinical trials conducted by their team with HLA-restricted GPC3 peptides indicating that these vaccines can improve prognosis without eliciting non-specific autoimmune responses in most HCC patients. Encouraging results with GPC3 peptide vaccine were observed in patients with HCC and in GPC3-positive advanced ovarian clear cell carcinoma in Phase II studies (Shimizu et al.).

GPC3 is highly expressed in a variety of pediatric solid embryonal cancers including hepatoblastomas, Wilms and rhabdoid tumors, germ cell tumor subtypes and a minority of rhabdomyosarcomas. Ortiz et al., highlight that, although clinical trials demonstrated the safety and potential benefit of GPC3-targeting strategies in adult patients, evaluation of these immunotherapies in pediatric patients may be more challenging considering the distinct physiological pattern of GPC3 expression in infants in liver and kidney. T cells genetically engineered with a GPC3-CAR (GAP T cells) and a GPC3 peptide vaccine are currently under clinical investigation in pediatric patients with GPC3-positive solid tumors. Next generation GPC3-targeting approaches such as TCR-engineered T cell therapy are also under development offering potential therapeutic options for these patients (Ortiz et al.).

In pancreatic cancer, GPC1 expression significantly correlates with pathologic grade and clinical stage, and is closely associated with poor prognosis. Wang et al., address mechanisms altering GPC1 expression in cancer including DNA hypomethylation, microRNA expression and KRAS mutation, and examine the role of glypican in mediating key cellular signaling in tumorigenesis and angiogenesis. The authors also review studies investigating the potential of circulating GPC1 as a cancer biomarker and discuss possible reasons that may account for the contradictory results reported previously (Wang et al.).

Sulfation degree and pattern heavily impact the HS interactive abilities and functions. Denys and Allain summarize the emerging evidence for a role of the 3-O-sulfotransferases (HS3STs) in cancer. By catalyzing glucosaminyl-3-O sulfation, they produce rare HS modifications which affect the selective binding of several ligands. Altered expression of HS3STs has been associated with tumor-promoting or tumor-repressing effects depending on cellular and environmental context, substrate specificity and subcellular distribution of the enzymes, availability of acceptors or compensatory expression of HS3ST isoenzymes. Epigenetic repression of the HS3ST2, HS3ST1, HS3ST3A1 family members was found to be associated with the progression of several malignancies. In contrast, overexpression of HS3ST3B promoted epithelial-to-mesenchymal transition and angiogenesis in pancreatic and lung cancer, likely by favoring ligand binding to cell surface HS and activation of NRP1, VEGF, and TGF-β signaling. The design of specific HS3ST inhibitors is expected to provide insights into the role of the enzymes in cancer and the opportunity of modulating HS 3-O-sulfation to improve therapies (Denys and Allain).

A challenging goal in this field is to determine how the HS glycoside sequence and sulfation pattern drive ligand binding specificity. Brunetti et al., address this issue using the tetrabranched peptide NT4 which selectively binds HS on the tumor surface. The authors found a correlation between NT4 cell binding and basal expression of Sulf-1 and−2, suggesting that peptide binding was affected by HS 6-O-sulfation. Moreover, investigation of structural determinants of HS binding sites suggested multivalent binding of NT4 to densely sulfated clusters and a higher affinity for GPC3 and GPC4 among HSPGs. In addition to be useful probes for structural studies, as cancer selective HS-targeted agents, NT4, and possibly newly designed peptides, exhibit a theranostic potential since they can be conjugated with various functional units for drug delivery or tracer transport for tumor imaging (Brunetti et al.).

Among HS modifying enzymes, heparanase, the only HS specific mammalian endo-β-D-glucuronidase, was the first to be investigated as a cancer drug target. Heparanase plays a well-recognized role in inflammation, tumorigenesis, cancer progression, and drug resistance. Coombe and Gandhi revisit the milestones of the heparanase discovery, re-examine its role as a cancer-associated and metastasis promoting enzyme, and discuss its multiple non-enzymatic activities in light of structural data. Early observation of the potent heparanase inhibitory activity of heparin paved the way for the screening of heparin/HS mimetics as heparanase inhibitors. Based on promising preclinical data, some of them are currently under clinical investigation although none has been approved yet. The authors comment on the difficulty of interpreting data with HS mimetics due to their pleiotropic effects including immunomodulation. The multifunctional activity of heparanase, its subcellular and extracellular localization and internalization mechanisms, as well as its contribution to physiological processes, are additional aspects that need to be clarified to fully understand the potential of heparanase, a valid but challenging target, according to the authors. They also point out the potential influence that the closely related heparanase 2 and the T5 heparanase splice variant, both lacking catalytic activity, may exert on *in vivo* efficacy of anti-heparanase drugs (Coombe and Gandhi).

An emerging area of clinical interest is the heparanase contribution to immune regulation. The production of heparanase by tumor and/or stromal cells (e.g., leukocytes) can result in mutual influence on gene expression and phenotypic behavior. The relative contribution of the enzyme from different cellular sources and the underlying molecular mechanisms are just beginning to be elucidated. Mayfosh et al., provide an overview of the current knowledge of heparanase expression and functions in leukocytes highlighting its two-sided role. Novel leukocyte-based anticancer therapies e.g., CAR-T cell therapy, dendritic cell vaccines and viral-therapeutic delivery exploiting heparanase are under development. The emerging picture is that the choice of the appropriate therapies inhibiting pro-tumorigenic or promoting anti-tumorigenic effects of heparanase will depend on a better understanding of the particular cancer setting. For instance, heparanase inhibitors may have more chance of being effective for malignancies in which leukocyte-derived heparanase promotes tumor progression such as colorectal and pancreatic carcinoma (Mayfosh et al.).

By applying CRISP-Cas9 technology and lentiviral cell infection to stably knock down or overexpress heparanase in colorectal cancer models, Liu et al., demonstrate that the endoglycosidase promoted tumor growth and liver metastatic dissemination. Transcriptome analysis confirmed the link between heparanase and genes/pathways involved in ECM remodeling. Among these, the metalloproteinase MMP1 was shown to be positively regulated by heparanase via p38 MAPK signaling (Liu et al.).

By using a mouse model of metabolic syndrome/diabetes and concurrent pancreatic ductal adenocarcinoma (PDAC), Goldberg et al., reveal a new mechanism underpinning the preferential heparanase overexpression in this malignancy. The study demonstrates that advanced glycation end-products (AGE), typical components of the diabetic milieu, induce heparanase expression in PDAC, suggesting that the endoglycosidase contributes to sustaining the known bidirectional relationship between diabetes and pancreatic tumorigenesis. The authors propose that heparanase may exacerbate PDAC-associated diabetes, further contributing to tumor progression and therapy resistance, and suggest that heparanase targeting approaches disrupting this reciprocal causality may provide clinical benefit in PDAC (Goldberg et al.).

Further insights into the role of heparanase in tumor progression are provided by Cohen-Kaplan et al. The authors show that heparanase-mediated activation of Src results in the phosphorylation of catenins with the consequent destabilization of the E-cadherin/catenin complex and disruption of adherent junctions. Reduced integrity of epithelial sheets, a feature associated with advanced tumor stages, represents an additional effect whereby heparanase, through a mechanism likely independent of enzymatic activity, promotes cancer cell migration (Cohen-Kaplan et al.).

Overall, investigation of HSPGs and their endogenous modifying enzymes reveals a highly complex system affecting tumor growth and progression, and continues to inspire novel anticancer strategies exploiting pro- or anti-tumorigenic effects of the various system components. Articles in this Research Topic show the potential of this lively field of research to indicate novel tumor markers and treatments to be explored in specific disease settings.

## Author Contributions

All authors listed have made a substantial, direct and intellectual contribution to the work, and approved it for publication.

### Conflict of Interest

The authors declare that the research was conducted in the absence of any commercial or financial relationships that could be construed as a potential conflict of interest.

